# A population‐based study to develop juvenile arthritis case definitions for administrative health data using model‐based dynamic classification

**DOI:** 10.1186/s12874-021-01296-9

**Published:** 2021-05-16

**Authors:** Allison Feely, Lily SH Lim, Depeng Jiang, Lisa M. Lix

**Affiliations:** 1 Department of Epidemiology and Cancer Registry, CancerCare Manitoba, Winnipeg, Canada; 2 Department of Paediatrics, Rady Faculty of Health Sciences, University of Manitoba, Winnipeg, Canada; 3 Department of Community Health Sciences, Rady Faculty of Health Sciences, University of Manitoba, S113-750 Bannatyne Avenue, R3E 0W3 Winnipeg, Canada

**Keywords:** Administrative data, Classification, Discriminant analysis, Longitudinal analyses, Juvenile arthritis

## Abstract

**Background:**

Previous research has shown that chronic disease case definitions constructed using population-based administrative health data may have low accuracy for ascertaining cases of episodic diseases such as rheumatoid arthritis, which are characterized by periods of good health followed by periods of illness. No studies have considered a dynamic approach that uses statistical (i.e., probability) models for repeated measures data to classify individuals into disease, non-disease, and indeterminate categories as an alternative to deterministic (i.e., non-probability) methods that use summary data for case ascertainment. The research objectives were to validate a model-based dynamic classification approach for ascertaining cases of juvenile arthritis (JA) from administrative data, and compare its performance with a deterministic approach for case ascertainment.

**Methods:**

The study cohort was comprised of JA cases and non-JA controls 16 years or younger identified from a pediatric clinical registry in the Canadian province of Manitoba and born between 1980 and 2002. Registry data were linked to hospital records and physician billing claims up to 2018. Longitudinal discriminant analysis (LoDA) models and dynamic classification were applied to annual healthcare utilization measures. The deterministic case definition was based on JA diagnoses in healthcare use data anytime between birth and age 16 years; it required one hospitalization ever or two physician visits. Case definitions based on model-based dynamic classification and deterministic approaches were assessed on sensitivity, specificity, and positive and negative predictive values (PPV, NPV). Mean time to classification was also measured for the former.

**Results:**

The cohort included 797 individuals; 386 (48.4 %) were JA cases. A model-based dynamic classification approach using an annual measure of any JA-related healthcare contact had sensitivity = 0.70 and PPV = 0.82. Mean classification time was 9.21 years. The deterministic case definition had sensitivity = 0.91 and PPV = 0.92.

**Conclusions:**

A model-based dynamic classification approach had lower accuracy for ascertaining JA cases than a deterministic approach. However, the dynamic approach required a shorter duration of time to produce a case definition with acceptable PPV. The choice of methods to construct case definitions and their performance may depend on the characteristics of the chronic disease under investigation.

**Supplementary Information:**

The online version contains supplementary material available at 10.1186/s12874-021-01296-9.

## Background

Administrative health data, which capture information about patient contacts with the health care system, are widely used for chronic disease research and surveillance [1–3]. Different approaches have been used to construct definitions to ascertain disease cases from these data. A common approach is to use a deterministic (i.e., non-probability) rule [4] applied to administrative health data; for example cases may be identified via any hospitalization with a disease-specific diagnosis code. Alternatively, model-based approaches apply statistical or machine-learning (i.e., probability) models to ascertain disease cases using multiple disease markers defined from administrative health data, including diagnoses, medication use, and healthcare procedures [5–10]. These model-based approaches have demonstrated greater accuracy than a deterministic case ascertainment approach for some chronic diseases, such as peripheral artery disease, hypertension, and kidney disease [7, 9, 10].

Model-based methods that consider the temporal aspects of healthcare contacts have also been proposed [11–15]. Recent studies have examined model-based dynamic classification [12, 16], which applies a statistical or machine-learning model to repeated measurements of disease-specific indicators. At each measurement occasion, individuals are classified into disease, non-disease, and indeterminate categories based on predicted probabilities of group membership; these model-based methods may require data from only a few measurement occasions to ascertain cases, and could therefore be more efficient (i.e., requiring a shorter duration of time) for making case ascertainment decisions. However, to date only a few studies have considered a model-based dynamic classification approach for disease case ascertainment [12, 16] and comparisons with deterministic case ascertainment methods have not been undertaken.

Our study purpose was to investigate the performance of a model-based dynamic classification approach to ascertain disease cases from administrative health data. The objectives were to validate a model-based dynamic classification approach for ascertaining cases of juvenile arthritis (JA) from administrative data, and compare the performance of a model-based dynamic classification approach with the performance of a deterministic approach for case ascertainment. We selected JA for this study because it is an episodic disease characterized by periods of good health followed by periods of illness that vary in severity and/or duration. We hypothesized that a case ascertainment approach that updates case status after each measurement occasion would produce accurate results in a shorter period of time than a deterministic case ascertainment method based on summary data. Moreover, we selected JA because validated, deterministic case definitions have already been developed, which provide a comparator for assessing the performance of the model-based dynamic classification approach.

## Methods

### Data Sources

This study was conducted using linked administrative health data and clinical registry data from the province of Manitoba, Canada for fiscal years 1980/81 to 2017/18 (a fiscal year extends from April 1 to March 31). Manitoba has a universal healthcare system; virtually all contacts with the healthcare system are captured for the entire population. Manitoba has a total population of approximately 1.3 million people according to Statistics Canada census data.

The administrative databases used in this study included the Manitoba Health Insurance Registry, Hospital Discharge Abstracts Database (DAD), and Medical Claims database; these data were used to ascertain disease cases. Pediatric rheumatology clinic data were linked to administrative data to define the study cohort and validate the case ascertainment methods. All databases were contained in the Manitoba Population Research Data Repository housed at the Manitoba Centre for Health Policy (MCHP), University of Manitoba, and were linked using a unique anonymized personal health identification number. Statistics Canada Census data for dissemination areas, the smallest geographic unit for which Census data are publically released, were used to define an area-level measure of socioeconomic status to describe the cohort.

The Manitoba Health Insurance Registry contains information about residents of Manitoba eligible for health insurance coverage; it captures dates of coverage, reasons for termination of coverage (e.g., migration out of province, death), and demographic characteristics (e.g., age, sex, residence location). The DAD contains diagnosis and procedure codes for patients discharged from Manitoba hospitals; diagnoses are recorded using the World Health Organization’s International Classification of Diseases (ICD). The 9th revision, clinical modification of ICD (i.e., ICD-9-CM) was used prior to April 1, 2004 and the 10th revision, Canadian version (i.e., ICD-10-CA) was used after this date; up to 25 diagnoses are captured on each record in the DAD. The Medical Claims database contains information on specialist and general practitioner (GP) physician billings for both in-hospital and outpatient visits; a single ICD-9-CM diagnosis is captured for each claim.

The Pediatric Rheumatology Clinical Database [17] captures diagnostic information for each child seen by a pediatric rheumatologist at the Children’s Hospital in Winnipeg, Manitoba. Given that there are no pediatric rheumatologists practicing outside of the Children’s Hospital in Winnipeg, this database captures diagnosed JA cases for the entire province. The registry includes clinically-confirmed diagnosis (i.e., type of JA diagnosis; non-JA diagnosis) and date of diagnosis.

### Study Cohort

The study cohort included Manitoba children in the Pediatric Rheumatology Clinical Database who were: (1) born between April 1, 1980 and March 31, 2002; (2) could be linked to the Manitoba Health Insurance Registry; (3) had a diagnosis recorded before age 16 years (i.e., the upper age limit for a clinical diagnosis of JA); (4) had continuous health insurance coverage in Manitoba from birth until age 16 years; and (5) had six-digit postal code recorded on their birth record to determine their residence location at birth. Individuals were born between April 1, 1980 and March 31, 2002 and were followed from birth until their 16th birthday in the years of administrative health data available for this study (i.e., April 1, 1980 to March 31, 2018).

The Pediatric Rheumatology Clinical Database includes JA cases as well as individuals for whom a JA diagnosis had been ruled out (i.e., non-JA controls), but who may have another condition diagnosed by a pediatric rheumatologist (e.g., musculoskeletal conditions, transient rheumatic conditions, systemic rheumatic or immune system conditions, rheumatic skin disease, uveitis). JA is defined as arthritis that begins before an individual’s 16th birthday and persists for at least six weeks [18]. Both the current juvenile idiopathic arthritis (JIA) disease classification [18] and the previous juvenile rheumatoid arthritis (JRA) disease classification [19] were used to identify JA cases. Specifically, patients diagnosed with JIA, JRA, and seronegative enthesopathy and arthropathy (SEA) syndrome were identified as JA cases.

### Study Variables

Four healthcare utilization variables were defined for the model-based dynamic classification approach (Table 1): any JA-related healthcare contact (binary variable), total number of GP physician visits (count variable), total number of specialist physician visits (count variable), and hospitalization for any reason (binary variable). These variables were selected based on existing JA case definitions [17, 20, 21]. Each variable was defined for each of 15 time periods; the first time period extended from birth to the second birthday (i.e. 0 and 1 years of age) and the remaining time periods each comprised a single year (i.e. 2, 3, 4, …, 15 years of age). The first time period was longer than then remaining time periods, because JA-specific healthcare use was infrequent in the first year of life, resulting in sparse cell counts.

**Table 1:** Healthcare utilization measures for model-based dynamic classification approach

Measure	Type	Data Source	Definition
Any JA-related healthcare contact	Binary	Medical Claims DAD	At least one JA-related diagnosis code (ICD-9-CM codes: 696, 713, 714, 716, 720; ICD-10-CA codes: M05-M09, M45)
Number of general practitioner visits	Count	Medical Claims	Total number of ambulatory visits to a general practitioner
Number of specialist visits	Count	Medical Claims	Total number of ambulatory visits to a specialist physician
Hospitalization	Binary	DAD	At least one hospitalization (excluding newborn hospitalization)

Note: *JA* juvenile arthritis; *DAD* Discharge Abstract Database; *ICD *International Classification of Diseases

Sociodemographic variables were also defined: sex, area of residence (urban, rural), age at diagnosis, time period of diagnosis, and income quintile. Sex and area of residence were defined from the Manitoba Health Insurance Registry; the latter was assigned based on six-digit postal code at birth. Urban residents were those who lived in Winnipeg, the largest city in Manitoba (population > 700,000); all others were rural. Age at diagnosis (1–5, 6–10 and 11–15 years) and time period of diagnosis (≤ 1992, 1993–2002, 2003–2012) were defined using date of diagnosis recorded in the Pediatric Rheumatology Clinical Database. Income quintile is an area-level measure of socio-economic status based on total household income from Statistics Canada Census data; it was defined by assigning individuals to income quintiles based on postal codes recorded in the insurance registry at birth [22, 23]. Individuals with a missing income quintile value were included in the lowest income quintile; they represented less than 0.5 % of the cohort.

### Case Ascertainment Methods

#### Deterministic Case Definition

The deterministic JA case definition was previously validated in Manitoba [17]. Individuals were classified as JA cases if, prior to the 16th birthday, they had one or more hospitalizations or two or more physician visits with a relevant diagnosis at least eight weeks apart but no more than two years apart. The relevant diagnosis codes are for rheumatoid arthritis and ankylosing spondylitis (ICD-9-CM codes: 714, 720; ICD-10-CA codes: M05, M06, M08, M45). Each cohort member’s hospital and physician records from birth to the 16th birthday were searched to assess whether the case definition criteria were met. In sensitivity analyses, we also applied the deterministic case definition to data from two shorter time periods: birth to 2 years of age and 14 to 16 years of age. These sensitivity analyses represent the situation where administrative data are only available for short periods of time, as is often the case when deterministic case definitions are validated. For this sensitivity analysis, individuals were only required to have continuous health coverage from birth to 2 years of age for the first analysis and from 14 to 16 years of age for the second analysis, respectively. Two-year time periods were chosen because the validated case definition requires two years of follow-up [17].

*Model-Based Case Definition Using Dynamic Classification.* The model-based dynamic classification approach used healthcare utilization accrued by a given time period and sociodemographic covariates (i.e., age, sex, area of residence) to predict group membership. The individual’s group membership probabilities were calculated at each consecutive measurement occasion using accrued information until the individual was classified into one of the groups based on a pre-defined allocation scheme. Once individuals were classified into one of the groups, their group membership probabilities were not updated further (i.e., their classification did not change) [12, 16].

Longitudinal discriminant analysis (LoDA) [12, 16] using multivariate generalized linear mixed models (MGLMM) was adopted to compute the group membership probabilities. Suppose that measurements for $R\ge 1$ healthcare utilization variables are available at multiple occasions for $N$ individuals, and that each individual belongs to one of $G$ groups; in this study$G$ = 2 (i.e., JA cases and non-JA controls). Let ${\mathbf{Y}}_{i,r}=\left({Y}_{i,r,1}, \dots , {Y}_{i,r,{n}_{r}}\right)$ denote the repeated measurements on the $r$^th^ variable for the $i$^th^ individual observed at time points ${\mathbf{t}}_{i,r}=\left({t}_{i,r,1},\dots ,{t}_{i,r,{n}_{r}}\right), {t}_{i,r,1}<\dots <{t}_{i,r,{n}_{r}}$. Denote ${\mathbf{v}}_{i,r,1},\dots , {\mathbf{v}}_{i,r,{n}_{r}}\in {\mathbb{R}}^{{p}_{r}}$ as vectors of covariates. The disease variables, measurement occasions, and covariates for the $i$^th^ individual are denoted by ${\mathbb{Y}}_{i}=\left({\mathbf{Y}}_{i,1},\dots , {\mathbf{Y}}_{i,R}\right)$ and ${\mathcal{C}}_{i}=\left({\mathbf{t}}_{i,1},\dots , {\mathbf{t}}_{i,R},{\mathbf{v}}_{i,\text{1,1}},\dots , {\mathbf{v}}_{i,R,{n}_{R}} \right)$. Given the $i$^th^ individual’s group status ${U}_{i}=g$ and latent random effects vector ${\mathbf{b}}_{i}=\left({\mathbf{b}}_{i,1},\dots , {\mathbf{b}}_{i,R}\right)$, the $j$^th^ repeated measurement ${Y}_{i,r,j} (j=1,\dots , {n}_{r})$ of the $r$^th^ longitudinal disease marker ($r=1,\dots , R$) is assumed to follow a distribution from an exponential family (e.g., normal, binomial, Poisson) with a dispersion parameter ${\varphi }_{r}^{g}$. The expectation is given by

1 $${h}_{r}^{-1}\left\{\text{E}\left(\left.{Y}_{i,r,j}\right|{\mathbf{b}}_{i},{U}_{i}=g\right)\right\}={\mathbf{x}}_{i,r,j}^{{g}^{\text{T}}}{\varvec{\upalpha }}_{r}^{g}+{\mathbf{z}}_{i,r,j}^{{g}^{\text{T}}}{\mathbf{b}}_{i,r},$$

where ${h}_{r}^{-1}$ is a chosen link function for the $r$^th^ longitudinal disease marker, ${\mathbf{x}}_{i,r,j}^{g}={\mathbf{x}}_{i,r,j}^{g}\left({\mathcal{C}}_{i}\right)$ and ${\mathbf{z}}_{i,r,j}^{g}={\mathbf{z}}_{i,r,j}^{g}\left({\mathcal{C}}_{i}\right)$ are vectors of covariates derived from the information in ${\mathcal{C}}_{i}$, and ${\varvec{\upalpha }}_{r}^{g}$ are the unknown fixed effects parameters. The dispersion parameter ${\varphi }_{r}^{g}$ is either known or unknown depending on the distribution of the $r$^th^ disease marker [12]

A Markov Chain Monte Carlo (MCMC)-based Bayesian estimation approach was used to estimate the unknown parameters of the MGLMM for each group [12, 16, 24]. The prior distribution of the model parameters was specified to be weakly informative, as per previous research [24]. The number of mixture components for the random effects distribution must be specified; the optimal number of mixture components can be determined by assessing model fit using the penalized expected deviance (PED) [25]. Lower values for the PED indicate better model fit. In this study, the number of mixture components was set to one for JA cases and non-JA controls, for a parsimonious model.

The MLGMMs were used to estimate group membership probabilities; Bayes theorem gives the probability that the individual belongs to group $g$ as

2 $${\mathcal{P}}_{g}=\frac{{\pi }_{g}{f}_{g}}{{\sum }_{\tilde{g}=0}^{G-1}{\pi }_{\tilde{g}}{f}_{\tilde{g}}} ,$$

where $${f}_{g}$$ is a predictive density for the individual’s observed longitudinal healthcare use given the model parameters and $${\pi }_{g}$$ is the prior probability of belonging to group $$g,$$$${\pi }_{\tilde{g}}$$ is the prior probability of belonging to each group ($$0<{\pi }_{\tilde{g}}<1, {\sum }_{\tilde{g}=0}^{G-1}{\pi }_{\tilde{g}}=1$$) and $${f}_{\tilde{g}}$$ is the predictive density for each group [26]. Naïve prior group probabilities of 0.50 for both JA cases and non-JA controls were adopted. The predictive density $${f}_{g}$$ in Eq. (2) can be specified using one of three prediction approaches: marginal, conditional, and random effects. In general, the accuracy of the prediction approach depends on the data being used and must be evaluated in the process of building and testing the model [26].

Once an individual’s group membership probabilities were computed, each individual was assigned to a group based on an *a priori* allocation scheme. The point nearest to the top left corner of the ROC curve, which minimizes ${d}^{2}={\left(1-Sensitivity\right)}^{2}+{\left(1-Specificity\right)}^{2}$, was chosen as the cut-off point. It has been previously shown in dynamic classification to be optimal for balancing sensitivity and specificity [16, 27]. Hughes et al. [16] developed an allocation scheme that incorporates the credible intervals (CrIs) of the group membership probabilities, to account for the variability between individuals in the uncertainty of these probabilities. The CrI allocation scheme may increase positive predictive value (PPV) without sacrificing sensitivity, specificity, and probability of correct classification (PCC). In this study, 99 % CrIs were adopted, as they were previously shown to perform optimally [16, 28]

### Statistical Analysis

JA cases and non-JA controls were described on the sociodemographic variables and healthcare utilization variables using means and standard deviations for continuous variables and frequencies and percentages for categorical variables. A correlation analysis was conducted to describe the relationship of the longitudinal outcomes across the time periods. Given that the longitudinal outcomes were comprised of both dichotomous and count variables, Spearman correlation coefficients were computed. Descriptive statistics were also produced for the deterministic case definition.

For the model-based dynamic classification approach, MGLMMs were fit separately to the data for JA cases and non-JA controls. Bernoulli models with logit link functions were fit for the binary variables and Poisson models with log link functions were fit for the count variables. A random intercept was included in all models to allow the means of the healthcare use variables to vary across individuals. Two models were fit to the data: (1) JA utilization model, which included a single healthcare utilization variable of any JA-related healthcare contact in addition to the sociodemographic covariates, and (2) full model, which included all four healthcare utilization variables in addition to the sociodemographic covariates. The models were fit using the MCMC algorithm developed by Komárek and Komárková [24] and the parameters of the models were described using their posterior means (i.e., means conditional on the data) and 95 % CrIs. The convergence of the MCMC algorithm was evaluated using trace plots, the Gelman-Rubin diagnostic [29] and auto-correlation plots.

The model-based dynamic classification approach was evaluated using five-fold cross-validation; all cohort members were randomly assigned to one of the five folds. For example, for fold 1, the MGLMMs were fit using data from folds 2 to 5 and then the predicted probabilities were used to classify each individual in fold 1 as a JA case, non-JA control, or indeterminate case. This process was repeated for the remaining folds. A confusion matrix that included the indeterminate category was created for each fold after the last time period. Classification accuracy measures were computed for each fold and then averaged across the five folds.

Classification accuracy measures included sensitivity, specificity, PPV, and negative predictive value (NPV) and their 95 % confidence intervals (95 % CIs). These measures were calculated for both the deterministic case definition and the model-based dynamic classification approach. As well, for the model-based dynamic classification approach, the proportion of indeterminate (i.e., unclassified) cases was produced. For the model-based dynamic classification approach, the classification accuracy measures were compared amongst the prediction approaches (i.e., marginal, conditional, and random effects) as well as between the two models. The best dynamic classification model was the model with the greatest area under the receiver operating characteristic curve (AUC). The classification accuracy measures were calculated for the models after each time period to describe the evolution of classification accuracy over time. As well, sensitivity and specificity were computed using the entire cohort, as well as only those cohort members who were classified (i.e., were not in the indeterminate category) at each measurement occasion.

## Results

### Description of Study Cohort

**Fig. 1:**
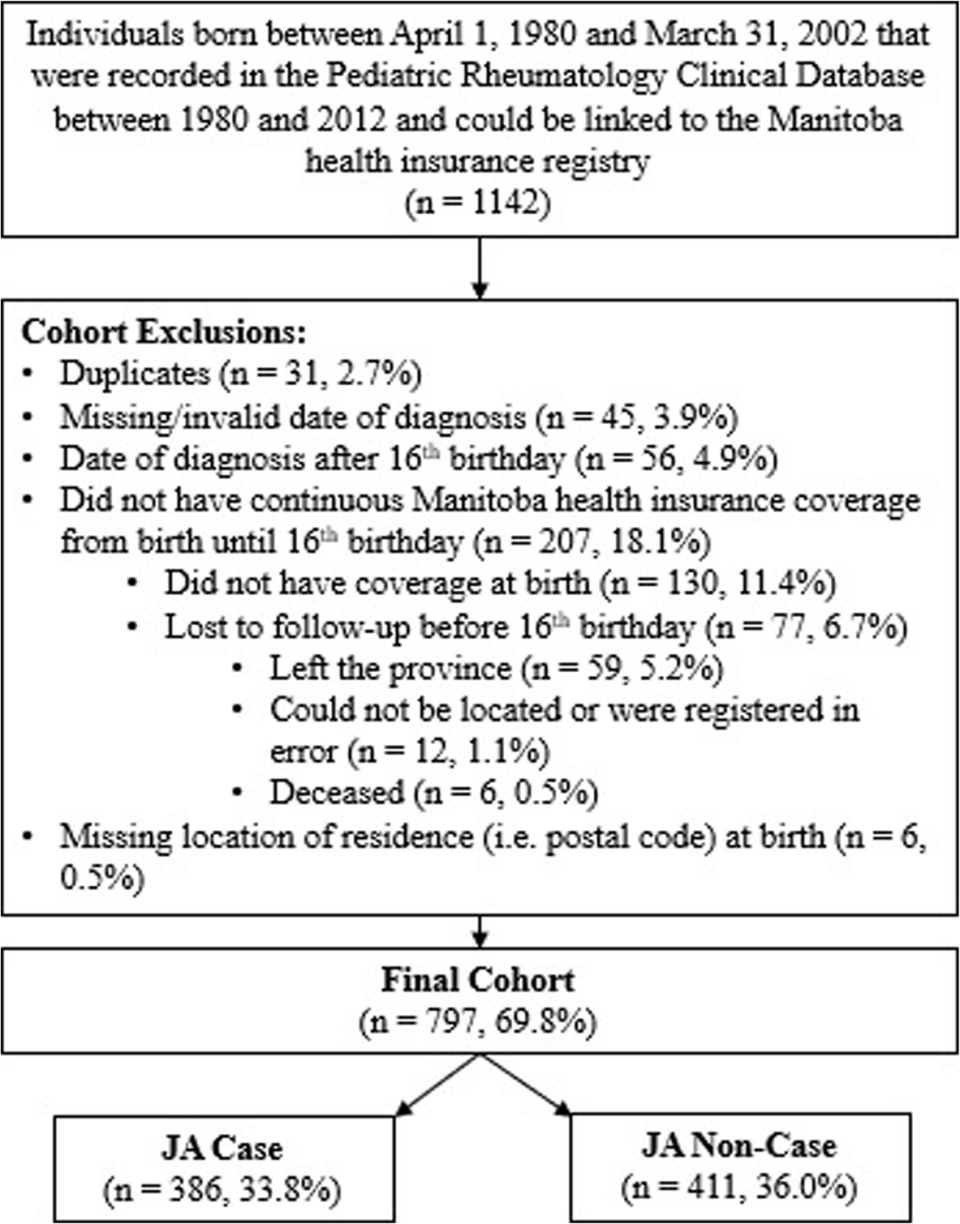
Study cohort flow chart

There were 1761 records in the Pediatric Rheumatology Clinical Database between 1980 and 2012; the years for which the database was constructed. A total of 1142 children captured in this database were born between April 1, 1980 and March 31, 2002. After applying other exclusion criteria, the final study cohort included 797 children of which 386 (48.4 %) were JA cases and 411 (51.6 %) were non-JA controls (Fig. 1). A total of 207 individuals were excluded because they did not have continuous Manitoba health insurance coverage between birth and their 16th birthday. Almost two-thirds (62.8 %) of these excluded individuals did not have health insurance coverage at birth, most often because they were not residents. The remaining individuals (*n* = 77) had health insurance coverage at birth but were lost to follow-up before their 16th birthday; three-quarters (76.6 %) of these individuals left the province, 15.6 % could not be located or were registered in error, and 7.8 % were deceased.

Characteristics of the study cohort stratified by JA cases and non-JA controls are provided in Table 2. Cohort members in both the JA case and non-JA control groups were more likely to be female (65.3 and 64.7 %, respectively), which was expected as pediatric rheumatic diseases are more common among girls [30, 31]. The distribution of age at diagnosis recorded in the clinical database was slightly different between the groups. Non-JA controls were more likely to be diagnosed at older ages, whereas cases were more likely to be diagnosed either in early childhood (i.e. 0–5 years of age) or early adolescence (i.e. 11–15 years of age). Period of diagnosis was dissimilar between the two groups, although the majority of cohort members in both the JA cases and non-JA control groups were diagnosed between 1993 and 2002 (49.7 and 79.3 %, respectively). Cohort members in both the JA case and non-JA control groups were more likely to live in an urban area at birth (56.5 and 57.9 %, respectively) and were generally evenly distributed across the income quintiles. There were no significant differences in income quintile between the two groups (*p*-value = 0.48). Characteristics of the study cohort stratified by JA case status and period of diagnosis are provided in Additional File 1.

**Table 2:** Characteristics of the study cohort, stratified by juvenile arthritis (JA) case status

Characteristic	JA Cases (*n* = 386)	Non-JA Controls (*n* = 411)	
	**n (%)**	**n (%)**	*P*-value*
Sex			
Male	134 (34.7)	145 (35.3)	0.87
Female	252 (65.3)	266 (64.7)	
Age at diagnosis (years)			
0–5	146 (37.8)	102 (24.8)	< 0.01
6–10	82 (21.2)	118 (28.7)	
11–15	158 (40.9)	191 (46.5)	
Period of diagnosis			
≤ 1992	83 (21.5)	6 (1.5)	< 0.01
1993–2002	192 (49.7)	326 (79.3)	
2003–2012	111 (28.8)	79 (19.2)	
Region of residence			
Urban	218 (56.5)	238 (57.9)	0.68
Rural	168 (43.5)	173 (42.1)	
Income quintile			
Q1 (Lowest/Not Found)	83 (21.5)	102 (24.8)	0.48
Q2	68 (17.6)	77 (18.7)	
Q3	80 (20.7)	67 (16.3)	
Q4	81 (21.0)	91 (22.1)	
Q5 (Highest)	74 (19.2)	74 (18.0)	
Any JA-related visit			
0–2 years	54 (14.0)	8 (1.9)	< 0.01
8 years	154 (39.9)	21 (5.1)	< 0.01
15 years	249 (64.5)	37 (9.0)	< 0.01
Number of general practitioner visits*			
0–2 years	10.51 (9.98)	9.35 (10.28)	0.11
8 years	1.64 (2.29)	1.86 (2.69)	0.22
15 years	2.17 (2.95)	2.05 (2.80)	0.56
Number of specialist visits*			
0–2 years	10.02 (11.38)	10.51 (11.33)	0.54
8 years	3.97 (4.86)	2.87 (4.89)	< 0.01
15 years	3.89 (4.10)	4.27 (5.98)	0.30
Hospitalization			
0–2 years	114 (29.5)	137 (33.3)	0.25
8 years	23 (6.0)	36 (8.8)	0.13
15 years	36 (9.3)	69 (16.8)	< 0.01

*Reported as mean (standard deviation); all other characteristics are reported as frequency (%)

As expected, JA cases were more likely to have a JA-related visit than non-JA controls at all ages. This likelihood increased consistently amongst cases over time (14.0-64.5 %). For controls, the likelihood of having a JA visit was low at all ages, but increased over time (1.9-9.0 %). For both the JA case and non-JA control groups, the mean number of GP physician visits was highest early in life (10.51 and 9.35, respectively). Similarly, the mean number of specialist physician visits was highest for both groups early in life (10.02 and 10.51). For the non-JA controls, the number of specialist physician visits was lower than for the JA cases until around age 10. Spearman correlation coefficients for each of the longitudinal outcomes across the time periods revealed moderate to high correlations amongst the earliest time periods, with a decay in correlation over time (see Additional File 1).

### Model‐Based Dynamic Classification Approach

The JA utilization model and full model were fit to the repeated measurements for the JA cases and non-JA controls. A summary of the estimated model parameters for these two models is provided in Table 3. The estimated model parameters for the fixed effects and the random intercept were similar for both the JA utilization model and the full model; only the results for the full model are described. For any JA visit, sex and age were statistically significant for JA cases; females were more likely to have a JA visit and age was positively associated with having a JA visit. For non-JA controls, region of residence and age were significantly associated with having a JA visit; children living in the urban region at birth were less likely to have a JA visit and age was positively associated with having a JA visit. For number of visits with a specialist physician, sex, region of residence and age were all statistically significant for JA cases; females, those living in the urban region at birth and younger age were associated with increased number of specialist visits. For non-JA controls, region of residence and age were significantly associated with the number of specialist physician visits; children living in the urban region at birth and younger ages were associated with an increased number of specialist visits. For both JA cases and non-JA controls, region of residence and age were negatively associated with hospitalization and the number of GP visits. The PED for the JA utilization model was 6168 and 2155 for JA cases and non-JA controls respectively.

**Table 3:** Posterior means (95 % credible intervals) for the JA utilization and full models

Variable	JA Utilization Model^a^		Full Model^b^	
	**JA Cases**	**Non-JA Controls**	**JA Cases**	**Non-JA Controls**
**JA-related visit**				
Male	**-0.60 (-0.97, -0.24)**	-0.07 (-0.44, 0.28)	**-0.59 (-0.95, -0.22)**	-0.07 (-0.43,0.27)
Urban	-0.24 (-0.59, 0.11)	**-0.57 (-0.91, -0.22)**	-0.24 (-0.60, 0.11)	**-0.53 (-0.86,-0.18)**
Age	**0.21 (0.19, 0.22)**	**0.17 (0.14, 0.20)**	**0.21 (0.19, 0.22)**	**0.17 (0.14,0.20)**
E(Intercept)	-1.76 (-2.09, -1.44)	-4.75 (-5.22, -4.28)	-1.77 (-2.11, -1.46)	-4.74 (-5.21,-4.28)
SD(Intercept)	1.59 (1.44, 1.75)	1.06 (0.86, 1.27)	1.60 (1.44, 1.75)	1.03 (0.84,1.23)
**# specialist visits**	--	--		
Male	--	--	**-0.19 (-0.37, -0.02)**	-0.13 (-0.32, 0.06)
Urban	--	--	**0.55 (0.38, 0.71)**	**0.63 (0.45, 0.81)**
Age	--	--	**-0.04 (-0.05, -0.04)**	**-0.03 (-0.04, -0.03)**
E(Intercept)	--	--	1.26 (1.12, 1.40)	0.91 (0.74, 1.07)
SD(Intercept)	--	--	0.80 (0.74, 0.86)	0.92 (0.85, 0.99)
**Hospitalization**	--	--		
Male	--	--	0.24 (-0.03, 0.52)	0.02 (-0.23, 0.25)
Urban	--	--	**-0.57 (-0.83, -0.32)**	**-0.58 (-0.80, -0.34)**
Age	--	--	**-0.11 (-0.13, -0.09)**	**-0.04 (-0.06, -0.03)**
E(Intercept)	--	--	-1.46 (-1.72, -1.20)	-1.47 (-1.71, -1.24)
SD(Intercept)	--	--	0.87 (0.73, 1.01)	0.84 (0.73, 0.96)
**# GP visits**	--	--		
Male	--	--	0.03 (-0.16, 0.24)	-0.02 (-0.22, 0.19)
Urban	--	--	**-0.50 (-0.68, -0.30)**	**-0.46 (-0.66, -0.25)**
Age	--	--	**-0.11 (-0.11, -0.10)**	**-0.10 (-0.10, -0.09)**
E(Intercept)	--	--	1.62 (1.46, 1.79)	1.45 (1.28, 1.63)
SD(Intercept)	--	--	0.94 (0.87, 1.02)	1.00 (0.92, 1.07)

Note: Values in boldface font indicate the fixed effect estimate was statistically significant at α = 0.05: *GP* general practitioner; *JA* juvenile arthritis; *E(Intercept)* mean of random intercept; *SD(Intercept)* standard deviation of random intercept

^a^JA utilization model contains one healthcare utilization variable: any JA-related healthcare contact; ^b^ Full model contains four healthcare utilization variables: (1) Any JA-related healthcare contact, (2) Number of specialist physician visits,(3) Hospitalization, (4) Number of general practitioner (GP) physician visits

For each of the models, visual assessment of the trace plots indicated that convergence was reach after the 1000th iteration. A total of 10,000 MCMC samples were drawn from the Gibbs sampler. The Gelman-Rubin diagnostic was used to ensure convergence to the target posterior distribution was reached. The upper confidence limits of the potential scale reduction factor (PSRF) were less than 1.02 for all parameters, suggesting convergence was reached with 10,000 samples. Autocorrelation plots indicated that the MCMC samples were correlated, thus, 1:100 thinning was applied. In summary, after determining convergence was reached by the 1000th iteration, the first 1000 samples were discarded as “burn-in” and the remaining 9000 samples were used for inference.

The predicted probabilities for the full model by JA case status and fold were summarized (see Additional File 1). For both the JA cases and non-JA controls, the means and standard deviations were similar across the five folds for the marginal and random effects prediction approaches. For both prediction approaches, the mean predicted probabilities increased over the time periods for the JA cases and decreased over the time periods for the non-JA controls. The standard deviations of the predicted probabilities increased over the time periods and were higher for the marginal prediction approach compared to the random effects approach.

**Fig. 2:**
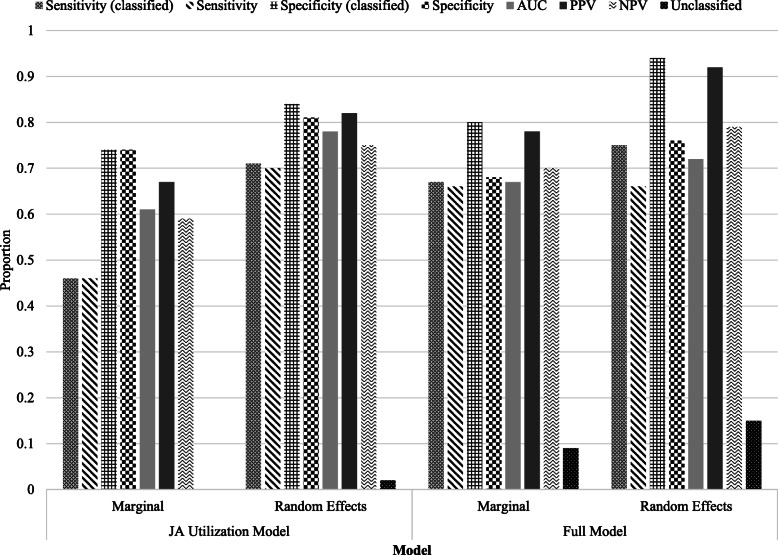
Accuracy measures for model-based dynamic classification approach for juvenile arthritis. Legend: Marginal and random effects prediction approaches were used for the JA utilization model and full model; AUC = area under the receiver operating characteristic curve; PPV = positive predictive value; NPV = negative predictive value

Figure 2 provides a summary of the classification accuracy for the JA utilization and full models for both the marginal and random effects predication approaches. The conditional prediction approach resulted in a similar fit to the marginal approach and its results are therefore not shown. For each model, the random effects prediction approach had higher classification accuracy than the marginal approach. However, the random effects prediction approach left more individuals unclassified after the last time period compared to the marginal approach. In addition, for both models, the random effects prediction had a higher mean classification time than the marginal approach, meaning, on average, it required more years of data to make a classification. On average, 6.44 (JA utilization model) and 2.78 (full model) additional years of data were needed to classify an individual as a JA case or non-JA control using the random effects approach.

For the marginal approach, the full model performed best on all classification accuracy measures, except for specificity, which was highest for the JA utilization model at 0.74. No individuals were left in the indeterminate category at the end of the measurement occasions using the JA utilization model. The proportion of indeterminate individuals for the full model was 0.09. Mean classification time was considerably lower for the JA utilization model at 2.77 years compared to 7.07 years for the full model.

For the random effects approach, sensitivity of the classified data ranged from 0.71 (JA utilization model) to 0.75 (full model) and was only slightly lower for all of the data, specificity of the classified data ranged from 0.84 (JA utilization model) to 0.94 (full model) and again was only slightly lower for all of the data, AUC ranged from 0.72 (full model) to 0.78 (JA model), PPV ranged from 0.82 (JA model) to 0.92 (full model), and NPV ranged from 0.75 (JA model) to 0.79 (full model). The proportion of individuals in the indeterminate category at the end of the observation period for the JA utilization and full models were 0.02 and 0.15, respectively. Mean classification time was similar for the JA utilization and full models at 9.21 and 9.85 years, respectively.

The JA utilization model using the random effects prediction approach was chosen as the best LoDA model, as it had the highest AUC amongst the models at 0.78. This model was used for the remaining analyses.

**Fig. 3:**
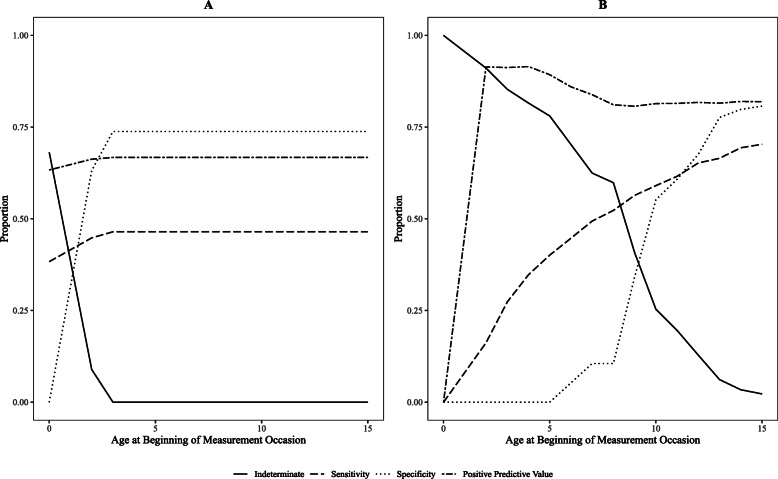
Proportion of indeterminate individuals, sensitivity, specificity, and positive predictive value for model-based dynamic classification approach. Legend: Based on the JA utilization model. Panel A shows results for the marginal prediction approach. Panel B shows results for the random effects approach

Figure 3 illustrates how the proportion of individuals unclassified, sensitivity, specificity, and PPV changed across measurement occasions for the model-based dynamic classification approach using the marginal and random effects prediction approaches for the JA utilization model. For the marginal approach, 68.1 % of individuals were left unclassified after the first measurement occasion (0 and 1 years of age), but dropped to only 8.9 % of individuals after the second measurement occasion (2 years of age). By the end of the third measurement occasion (3 years of age), all cohort members were classified. Accordingly, the accuracy measures changed during the first three measurement occasions and then remained constant, as there were no new individuals to be classified. For the random effects approach, all individuals were left unclassified after the first measurement occasion. The proportion of unclassified individuals then steadily decreased to 0.02 after the 15th measurement occasion (15 years of age). Sensitivity increased across all the time periods, to 0.70 after the last measurement occasion, whereas specificity remained at 0 until after the fifth measurement occasion (i.e., 5 years of age) where it increased to 0.81 after the last measurement occasion. PPV increased to 0.91 and then slightly decreased to 0.82.

**Fig. 4:**
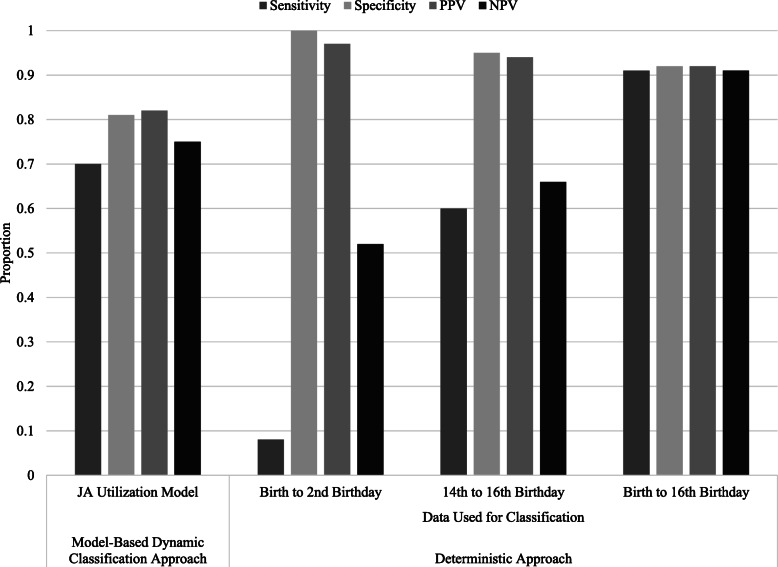
Accuracy of the model-based dynamic classification approach and deterministic approach for juvenile arthritis case definitions. Legend: PPV = positive predictive value; NPV = negative predictive value

Figure 4 summarizes the classification accuracy of the deterministic case definition. The case definition that used data from all 15 measurement occasions (i.e. birth to 16th birthday) had sensitivity of 0.91, specificity of 0.92, PPV of 0.92, and NPV of 0.91. When this deterministic case definition was applied to two-year intervals (i.e., birth to 2nd birthday, 14th to 16th birthdays), sensitivity and NPV were lower than when the case definition was applied to all years of data. The case definitions applied to the two-year intervals achieved higher specificity and PPV than the case definition applied to all years of data.

The model-based dynamic classification approach did not outperform the deterministic approach that used all 15 repeated measurements on any of the classification accuracy measures. The case definition for the model-based dynamic classification approach resulted in a sensitivity estimate that was 23.1 % lower than the sensitivity of the deterministic case definition. The dynamic model did, however, achieve higher sensitivity and NPV than the deterministic case definition when the latter was applied to just two years of data.

## Discussion

In this study, a model-based dynamic classification approach was applied to Manitoba’s administrative health data linked to a clinical registry comprised of JA cases and non-JA controls. Classification accuracy of the dynamic classification approach was compared to a validated deterministic case definition [17]. Based on the AUC, the JA utilization model using the random effects prediction approach was chosen as the best dynamic classification model. The dynamic classification model had lower accuracy for ascertaining JA cases than the deterministic case definition. However, the dynamic classification approach required a shorter duration to produce a case definition with acceptable PPV.

Although the model-based dynamic classification approach did not outperform the conventional deterministic case definition [17] in this application, there are other chronic diseases where this may not be the case. Specifically, chronic conditions that are episodic in nature and have changing patterns of healthcare utilization over time, such as inflammatory bowel disease [32], may benefit from the use of a dynamic classification approach to construct case definitions.

In this study, a chronic disease with well-defined age limits for diagnosis (i.e., birth to 16th birthday) [18] was selected for investigation. This will not be the case for all chronic conditions, especially those that are likely to have an age of onset in adulthood; it may not make sense or be possible to use an individual’s complete longitudinal health history from birth. The observation period for applying a model-based dynamic classification approach may be defined using a defined period of time following a specific treatment or procedure date [8, 10, 28, 33]. A specified number of years or an age range could also be used to define the observation period [11].

In addition, the timing of classification updates needs to be considered. In this study, the first classification was conducted at the second birthday using data accrued from birth, and then the classifications were updated annually until the 16th birthday. However, the classifications could have been updated with other time intervals/frequencies, depending on the nature of the disease under study and the probability of disease onset at different ages. An individualized updating schedule, similar to that used in previous applications of the model-based dynamic classification approach [12, 16, 28] could also be used. An individual’s classification is then updated each time a new health care visit is recorded in administrative health data. Researchers must carefully consider the optimal updating approach based on the features of the data available to them and the characteristics of the health condition of interest. Implementation of the model-based dynamic classification approach also requires consideration of the longitudinal disease markers of interest, additional model covariates, and random effects. In this study, the disease markers and covariates were chosen based on prior research [31, 34, 35]. Fit statistics may also be used to provide empirical evidence about the choices to be made.

One limitation of the model-based dynamic classification approach is that once an individual is classified, their status is not revisited at subsequent measurement occasions. For example, if an individual had a high probability of not being a JA case earlier in their life and was classified as a non-JA control, their status was not revisited later in time. This may result in misclassification for diseases that evolve over time, that is, where the measures used for classification show changes in their trends or associations with one another over time. However, no studies to our knowledge have examined the impact of updating the predicted probabilities on misclassification bias. Another limitation was that only one updating approach was used to make classifications. Applying a different updating approach to the study data, such as updating the results each time a new visit was recorded in the administrative health data, could influence the final classification results. Another potential limitation is that a normal distribution was chosen for the random effects in the MGLMMs, instead of a more flexible mixture normal distribution. If the random effects distribution is misspecified, there is potential for the estimates of the model parameters to be biased and this may affect the performance of the discriminant analysis, although this impact was expected to be negligible based on previous research [12]. Finally, the study cohort was a referral population defined from the Pediatric Rheumatology Clinical Database, which had a higher percentage of JA cases than what would be seen in the general population. This may affect the generalizability of the results to the study population, an issue that has arisen in similar studies in which clinical registry data are used for case definition validation [17, 20].

## Conclusions

In summary, this study suggests that a model-based dynamic classification approach can produce chronic disease case definitions using longitudinal information from administrative health data with acceptable PPV. However, the PPV from the dynamic classification model was lower than the PPV produced by the deterministic case definition. In this disease setting, the deterministic case definition ascertained JA cases with greater accuracy than the dynamic classification model. However, there can be value associated with using longitudinal information in a dynamic manner to construct case definitions, because the evolution of the classification process can be described. Currently, the deterministic approach is the most widely-used approach to construct case definitions for population-based chronic disease research and surveillance [4, 17, 36]. However, researchers need to determine the implications of classifying an individual as a disease case or non-disease control if there is insufficient evidence to do so. Dynamic classification allows individuals to remain unclassified if specified criteria indicate that more information is needed to make a potentially more accurate decision.

Instead of viewing case definition development as “one approach fits all”, researchers should carefully consider the characteristics of their disease of interest, as well as the data available, to determine which approach, deterministic, model-based, or dynamic, may result in the greatest classification accuracy. The choice of methods to construct chronic disease case definitions and their performance will depend on the characteristics of the disease of interest.

## Supplementary Information

**Additional file 1: **Dynamic Classification Results. This file includes three tables and one figure that contain: (a) descriptive characteristics of the study cohort stratified by period of diagnosis, (b) Spearman correlation coefficients for the outcome variables across the time periods of measurement, and (c) predicted probabilities of being a Juvenile Arthritis (JA) case for selected models.

## Data Availability

The data that support the findings of this study are not publicly available. The data that support the findings of this study are available, with submission of appropriate data access approvals and ethics approval, from the Manitoba Centre for Health Policy at the University of Manitoba. Data access approvals are given by the Manitoba Health Information Privacy Committee and the Winnipeg Regional Health Authority upon application receipt and review. Ethics approval is given by the University of Manitoba Health Research Ethics Board.

## Abbreviations

AUC: Area under the curve
CrI: Credible interval
DAD: Discharge Abstract Database
ICD-10-CA: International Classification of Diseases, Tenth Revision, Canada
ICD-9-CM: International Classification of Diseases, Ninth Revision, Clinical Modification
JA: Juvenile arthritis
JIA: Juvenile idiopathic arthritis
JRA: Juvenile rheumatoid arthritis
LoDA: Longitudinal discriminant analysis
MCHP: Manitoba Centre for Health Policy
MCMC: Markov chain Monte Carlo
MGLMM: Multivariate generalized linear mixed model
NPV: Negative predictive value
PCC: Probability of correct classification
PED: Penalized expected deviance
PPV: Positive predictive value
PSRF: Potential scale reduction factor
ROC: Receiver operating characteristic curve
SEA: Seronegative enthesopathy and arthropathy syndrome
