# Supplementary material for: A population‐based study to develop juvenile arthritis case definitions for administrative health data using model‐based dynamic classification
Source: BMC Med Res Methodol. 2021 May 16;21:105. doi: 10.1186/s12874-021-01296-9 (PMC8127203; doi:10.1186/s12874-021-01296-9)

Additional File: Supplementary Tables and Figures

Table 1: Characteristics of the study cohort, stratified by juvenile arthritis (JA) case status and period of diagnosis

	**Period of Diagnosis**			
	**≤ 2002[a]**		**2003–2012**	
	**JA Cases (n = 275)**	**Non-JA Controls (n = 332)**	**JA Cases (n = 111)**	**Non-JA Controls (n = 79)**
**Characteristic**	**n (%)**	**n (%)**	**n (%)**	**n (%)**
Sex				
Male	94 (34.2)	125 (37.6)	40 (36.0)	20 (25.3)
Female	181 (65.8)	207 (62.4)	71 (64.0)	59 (74.7)
Age at diagnosis (years)				
0–10	188 (68.4)	188 (56.6)	40 (36.0)	32 (40.5)
11–15	87 (31.6)	144 (43.4)	71 (64.0)	47 (59.5)
Region of residence				
Urban	152 (55.3)	200 (60.2)	66 (59.5)	38 (48.1)
Rural	123 (44.7)	132 (39.8)	45 (40.5)	41 (51.9)
Income Quintile				
Q1 (Lowest/Not Found)	58 (21.1)	79 (23.8)	25 (22.5)	23 (29.1)
Q2	50 (18.2)	60 (18.1)	18 (16.2)	17 (21.5)
Q3	56 (20.4)	53 (16.0)	24 (21.6)	14 (17.7)
Q4	55 (20.0)	74 (22.3)	26 (23.4)	17 (21.5)
Q5 (Highest)	56 (20.4)	66 (19.9)	18 (16.2)	8 (10.1)

[a] The ≤1992 and 1993-2002 period of diagnosis groups were combined due to small (< 5) cell sizes in the ≤1992 period of diagnosis group

Table 2: Spearman correlation coefficients for the outcome variables across time periods

A) Any JA-related healthcare contact

Time Period	1	2	3	4	5	6	7	8	9	10	11	12	13	14	15
1	1.00														
2	0.64	1.00													
3	0.52	0.76	1.00												
4	0.44	0.66	0.85	1.00											
5	0.40	0.53	0.68	0.78	1.00										
6	0.33	0.47	0.61	0.68	0.77	1.00									
7	0.33	0.46	0.53	0.61	0.68	0.77	1.00								
8	0.25	0.33	0.42	0.50	0.60	0.65	0.74	1.00							
9	0.27	0.34	0.43	0.47	0.53	0.57	0.67	0.70	1.00						
10	0.19	0.30	0.34	0.38	0.45	0.53	0.58	0.59	0.73	1.00					
11	0.20	0.22	0.27	0.31	0.36	0.41	0.46	0.48	0.57	0.65	1.00				
12	0.17	0.15	0.17	0.22	0.26	0.30	0.36	0.37	0.47	0.55	0.70	1.00			
13	0.17	0.13	0.19	0.20	0.23	0.28	0.29	0.28	0.35	0.41	0.57	0.68	1.00		
14	0.13	0.12	0.13	0.15	0.18	0.22	0.25	0.24	0.27	0.34	0.48	0.53	0.68	1.00	
15	0.10	0.10	0.11	0.13	0.17	0.22	0.25	0.22	0.26	0.32	0.41	0.46	0.59	0.69	1.00

B) Number of general practitioner (GP) visits

Time Period	1	2	3	4	5	6	7	8	9	10	11	12	13	14	15
1	1.00														
2	0.71	1.00													
3	0.60	0.64	1.00												
4	0.60	0.61	0.62	1.00											
5	0.58	0.57	0.58	0.63	1.00										
6	0.49	0.49	0.53	0.57	0.65	1.00									
7	0.43	0.42	0.46	0.52	0.53	0.57	1.00								
8	0.37	0.39	0.46	0.45	0.50	0.50	0.57	1.00							
9	0.35	0.37	0.40	0.43	0.44	0.44	0.51	0.52	1.00						
10	0.32	0.31	0.34	0.40	0.40	0.40	0.43	0.46	0.50	1.00					
11	0.33	0.35	0.40	0.42	0.41	0.44	0.46	0.46	0.51	0.51	1.00				
12	0.26	0.29	0.33	0.31	0.34	0.35	0.34	0.37	0.40	0.45	0.51	1.00			
13	0.27	0.29	0.30	0.28	0.35	0.35	0.34	0.35	0.40	0.47	0.42	0.55	1.00		
14	0.20	0.23	0.20	0.23	0.26	0.27	0.27	0.29	0.27	0.34	0.37	0.47	0.53	1.00	
15	0.25	0.24	0.26	0.26	0.28	0.30	0.26	0.30	0.30	0.40	0.34	0.41	0.46	0.48	1.00

C) Number of specialist physician visits

Time Period	1	2	3	4	5	6	7	8	9	10	11	12	13	14	15
1	1.00														
2	0.69	1.00													
3	0.61	0.73	1.00												
4	0.56	0.63	0.73	1.00											
5	0.53	0.57	0.64	0.76	1.00										
6	0.46	0.53	0.57	0.65	0.76	1.00									
7	0.42	0.48	0.51	0.59	0.67	0.72	1.00								
8	0.34	0.39	0.42	0.50	0.56	0.60	0.72	1.00							
9	0.36	0.36	0.39	0.44	0.48	0.54	0.60	0.74	1.00						
10	0.26	0.26	0.29	0.34	0.38	0.40	0.49	0.57	0.71	1.00					
11	0.27	0.28	0.27	0.31	0.34	0.37	0.43	0.49	0.57	0.64	1.00				
12	0.20	0.21	0.22	0.23	0.25	0.28	0.34	0.36	0.45	0.54	0.67	1.00			
13	0.21	0.19	0.19	0.19	0.21	0.26	0.31	0.33	0.40	0.43	0.54	0.70	1.00		
14	0.20	0.19	0.17	0.17	0.19	0.21	0.21	0.24	0.30	0.30	0.42	0.50	0.64	1.00	
15	0.17	0.14	0.13	0.10	0.13	0.14	0.16	0.18	0.23	0.27	0.35	0.43	0.49	0.64	1.00

D) Hospitalization

Time Period	1	2	3	4	5	6	7	8	9	10	11	12	13	14	15
1	1.00														
2	0.23	1.00													
3	0.16	0.17	1.00												
4	0.08	0.14	0.15	1.00											
5	0.01	0.02	0.08	0.10	1.00										
6	0.12	0.15	0.14	0.12	0.16	1.00									
7	0.08	0.05	0.10	0.12	0.09	0.14	1.00								
8	0.07	0.16	0.12	0.11	0.08	0.16	0.16	1.00							
9	0.07	0.03	0.09	0.08	0.07	0.10	0.11	0.18	1.00						
10	0.08	0.08	0.12	0.09	0.06	0.06	0.08	0.16	0.23	1.00					
11	0.05	0.07	0.09	0.06	0.01	0.08	0.06	0.07	0.20	0.20	1.00				
12	0.10	0.08	0.06	0.03	0.02	0.09	0.09	0.05	0.11	0.12	0.19	1.00			
13	0.06	0.07	0.05	0.02	0.01	0.04	0.07	0.12	0.11	0.16	0.14	0.27	1.00		
14	0.08	0.10	0.10	0.05	0.06	0.03	0.10	0.06	0.11	0.17	0.21	0.24	0.37	1.00	
15	0.12	0.04	0.10	-0.01	0.00	0.05	0.11	0.02	0.09	0.03	0.06	0.12	0.15	0.23	1.00

Table 3: Mean (standard deviation) for predicted probabilities of being a Juvenile Arthritis (JA) case for the longitudinal discriminant analysis over the time periods using the full model, by JA status and fold

A) Marginal Prediction Approach

Time	JA Cases						Non-JA Controls					
Period	Fold 1	Fold 2	Fold 3	Fold 4	Fold 5	Overall	Fold 1	Fold 2	Fold 3	Fold 4	Fold 5	Overall
1	0.57	0.50	0.53	0.48	0.51	0.52	0.47	0.46	0.45	0.48	0.44	0.46
	(0.21)	(0.17)	(0.20)	(0.20)	(0.18)	(0.19)	(0.10)	(0.11)	(0.07)	(0.12)	(0.10)	(0.10)
2	0.60	0.51	0.54	0.53	0.51	0.54	0.45	0.43	0.42	0.46	0.41	0.43
	(0.26)	(0.21)	(0.25)	(0.26)	(0.23)	(0.24)	(0.11)	(0.12)	(0.11)	(0.14)	(0.12)	(0.12)
3	0.65	0.55	0.54	0.58	0.52	0.57	0.42	0.41	0.40	0.44	0.40	0.41
	(0.29)	(0.25)	(0.27)	(0.29)	(0.26)	(0.27)	(0.12)	(0.14)	(0.12)	(0.14)	(0.15)	(0.13)
4	0.65	0.57	0.54	0.62	0.54	0.58	0.42	0.38	0.38	0.43	0.39	0.40
	(0.31)	(0.28)	(0.28)	(0.30)	(0.28)	(0.29)	(0.14)	(0.14)	(0.12)	(0.16)	(0.16)	(0.14)
5	0.66	0.59	0.56	0.64	0.58	0.61	0.41	0.38	0.36	0.42	0.40	0.39
	(0.33)	(0.30)	(0.30)	(0.32)	(0.30)	(0.31)	(0.16)	(0.17)	(0.13)	(0.17)	(0.19)	(0.16)
6	0.66	0.60	0.59	0.66	0.61	0.62	0.41	0.38	0.34	0.42	0.41	0.39
	(0.34)	(0.32)	(0.31)	(0.32)	(0.31)	(0.32)	(0.17)	(0.18)	(0.13)	(0.20)	(0.21)	(0.18)
7	0.69	0.63	0.61	0.68	0.63	0.65	0.40	0.37	0.34	0.41	0.42	0.39
	(0.34)	(0.32)	(0.32)	(0.32)	(0.32)	(0.32)	(0.18)	(0.20)	(0.17)	(0.21)	(0.22)	(0.20)
8	0.71	0.65	0.64	0.70	0.65	0.67	0.40	0.37	0.34	0.42	0.42	0.39
	(0.34)	(0.32)	(0.32)	(0.31)	(0.32)	(0.32)	(0.20)	(0.21)	(0.18)	(0.22)	(0.23)	(0.21)
9	0.72	0.68	0.65	0.73	0.68	0.69	0.39	0.35	0.34	0.39	0.42	0.38
	(0.34)	(0.31)	(0.33)	(0.32)	(0.32)	(0.32)	(0.22)	(0.21)	(0.19)	(0.22)	(0.24)	(0.22)
10	0.72	0.70	0.66	0.75	0.70	0.71	0.37	0.33	0.32	0.39	0.42	0.37
	(0.35)	(0.32)	(0.34)	(0.32)	(0.33)	(0.33)	(0.24)	(0.22)	(0.19)	(0.23)	(0.27)	(0.23)
11	0.74	0.73	0.69	0.78	0.72	0.73	0.35	0.31	0.31	0.38	0.40	0.35
	(0.34)	(0.31)	(0.33)	(0.30)	(0.32)	(0.32)	(0.25)	(0.22)	(0.22)	(0.24)	(0.27)	(0.24)
12	0.76	0.75	0.73	0.81	0.74	0.76	0.34	0.30	0.29	0.35	0.39	0.33
	(0.34)	(0.30)	(0.31)	(0.28)	(0.32)	(0.31)	(0.24)	(0.24)	(0.24)	(0.26)	(0.29)	(0.25)
13	0.78	0.78	0.78	0.84	0.75	0.79	0.31	0.27	0.27	0.34	0.37	0.31
	(0.32)	(0.28)	(0.29)	(0.27)	(0.31)	(0.29)	(0.25)	(0.24)	(0.26)	(0.26)	(0.30)	(0.26)

14	0.79	0.81	0.81	0.85	0.76	0.80	0.29	0.24	0.26	0.32	0.34	0.29
	(0.31)	(0.27)	(0.27)	(0.26)	(0.32)	(0.29)	(0.25)	(0.23)	(0.27)	(0.27)	(0.30)	(0.26)
15	0.80	0.81	0.82	0.85	0.77	0.81	0.25	0.21	0.24	0.29	0.30	0.26
	(0.29)	(0.26)	(0.27)	(0.26)	(0.31)	(0.28)	(0.26)	(0.23)	(0.27)	(0.28)	(0.30)	(0.27)

B) Random Effects Prediction Approach

**Time**	**JA Cases**						**Non-JA Controls**					
**Period**	**Fold 1**	**Fold 2**	**Fold 3**	**Fold 4**	**Fold 5**	**Overall**	**Fold 1**	**Fold 2**	**Fold 3**	**Fold 4**	**Fold 5**	**Overall**
1	0.50	0.46	0.50	0.43	0.51	0.48	0.49	0.45	0.50	0.45	0.49	0.48
	(0.05)	(0.06)	(0.05)	(0.08)	(0.07)	(0.06)	(0.04)	(0.05)	(0.03)	(0.06)	(0.06)	(0.05)
2	0.53	0.45	0.50	0.44	0.49	0.48	0.46	0.42	0.46	0.43	0.45	0.44
	(0.12)	(0.12)	(0.10)	(0.12)	(0.12)	(0.12)	(0.05)	(0.06)	(0.04)	(0.07)	(0.06)	(0.06)
3	0.57	0.47	0.50	0.48	0.49	0.50	0.43	0.39	0.43	0.41	0.41	0.41
	(0.20)	(0.18)	(0.16)	(0.20)	(0.17)	(0.18)	(0.06)	(0.08)	(0.05)	(0.08)	(0.07)	(0.07)
4	0.60	0.51	0.52	0.53	0.50	0.53	0.42	0.37	0.41	0.39	0.38	0.39
	(0.25)	(0.23)	(0.21)	(0.25)	(0.22)	(0.23)	(0.07)	(0.08)	(0.06)	(0.09)	(0.07)	(0.07)
5	0.62	0.53	0.53	0.56	0.52	0.55	0.40	0.35	0.39	0.38	0.37	0.38
	(0.28)	(0.26)	(0.24)	(0.28)	(0.24)	(0.26)	(0.07)	(0.08)	(0.06)	(0.09)	(0.08)	(0.08)
6	0.63	0.55	0.54	0.58	0.55	0.57	0.38	0.34	0.37	0.36	0.36	0.36
	(0.30)	(0.28)	(0.27)	(0.30)	(0.27)	(0.28)	(0.08)	(0.09)	(0.06)	(0.10)	(0.10)	(0.09)
7	0.64	0.57	0.55	0.60	0.57	0.59	0.37	0.32	0.35	0.35	0.35	0.35
	(0.31)	(0.30)	(0.28)	(0.31)	(0.29)	(0.30)	(0.08)	(0.10)	(0.07)	(0.12)	(0.10)	(0.09)
8	0.65	0.58	0.57	0.62	0.59	0.60	0.36	0.31	0.33	0.35	0.33	0.34
	(0.32)	(0.31)	(0.30)	(0.32)	(0.31)	(0.31)	(0.10)	(0.10)	(0.08)	(0.12)	(0.10)	(0.10)
9	0.66	0.59	0.58	0.65	0.61	0.32	0.35	0.30	0.32	0.33	0.32	0.32
	(0.33)	(0.31)	(0.31)	(0.32)	(0.32)	(0.32)	(0.11)	(0.10)	(0.09)	(0.13)	(0.11)	(0.11)
10	0.67	0.61	0.59	0.67	0.63	0.63	0.34	0.28	0.31	0.32	0.31	0.31
	(0.33)	(0.32)	(0.32)	(0.32)	(0.32)	(0.32)	(0.12)	(0.10)	(0.10)	(0.13)	(0.12)	(0.11)
11	0.69	0.64	0.61	0.69	0.65	0.66	0.32	0.26	0.29	0.31	0.30	0.30
	(0.32)	(0.31)	(0.32)	(0.31)	(0.32)	(0.32)	(0.12)	(0.11)	(0.11)	(0.14)	(0.13)	(0.12)
12	0.70	0.66	0.63	0.73	0.68	0.68	0.31	0.26	0.28	0.30	0.29	0.29
	(0.32)	(0.31)	(0.31)	(0.30)	(0.31)	(0.31)	(0.13)	(0.12)	(0.12)	(0.15)	(0.13)	(0.13)

13	0.73	0.69	0.67	0.76	0.70	0.71	0.30	0.24	0.27	0.28	0.27	0.27
	(0.30)	(0.29)	(0.30)	(0.28)	(0.30)	(0.29)	(0.14)	(0.12)	(0.13)	(0.14)	(0.13)	(0.13)
14	0.76	0.72	0.71	0.78	0.72	0.74	0.28	0.23	0.26	0.27	0.25	0.26
	(0.28)	(0.27)	(0.28)	(0.27)	(0.29)	(0.28)	(0.15)	(0.12)	(0.15)	(0.15)	(0.14)	(0.14)
15	0.79	0.74	0.73	0.79	0.74	0.76	0.27	0.20	0.24	0.26	0.23	0.24
	(0.24)	(0.25)	(0.27)	(0.26)	(0.27)	(0.26)	(0.16)	(0.13)	(0.16)	(0.16)	(0.14)	(0.15)

Figure 1: Mean predicted probabilities of being a Juvenile Arthritis (JA) case for the longitudinal discriminant analysis over the time periods using the full model averaged across folds, by JA status

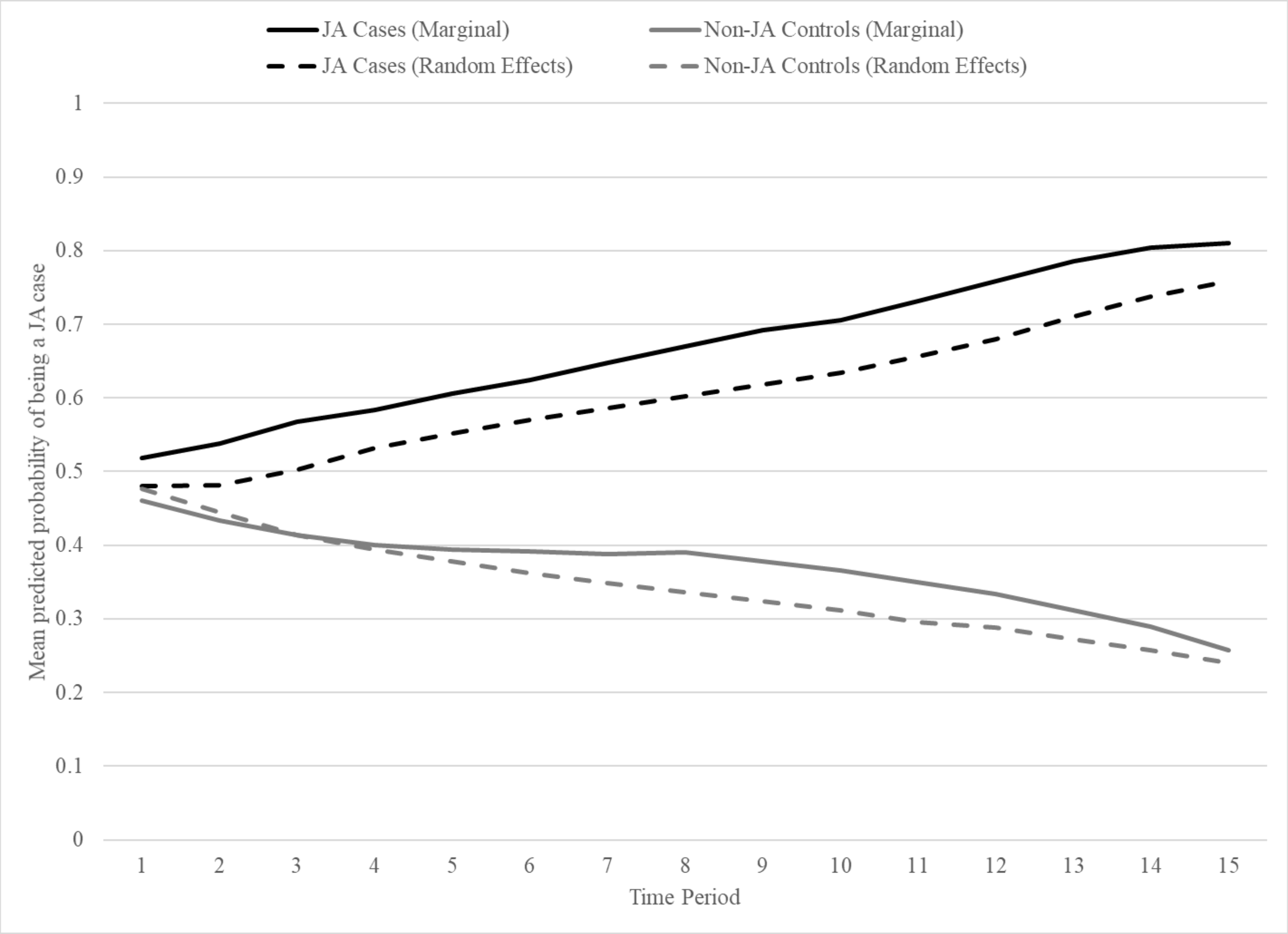

Supplement: Supplementary file 1 — Additional file 1: Dynamic Classification Results. This file includes three tables and one figure that contain: (a) descriptive characteristics of the study cohort stratified by period of diagnosis, (b) Spearman correlation coefficients for the outcome variables across the time periods of measurement, and (c) predicted probabilities of being a Juvenile Arthritis (JA) case for selected models. [file 12874_2021_1296_MOESM1_ESM.pdf]
